# Estrogen-Receptor-Low-Positive Breast Cancer: Pathological and Clinical Perspectives

**DOI:** 10.3390/curroncol30110706

**Published:** 2023-11-04

**Authors:** Christina Panagiotis Malainou, Nikolina Stachika, Aikaterini Konstantina Damianou, Aristotelis Anastopoulos, Ioanna Ploumaki, Efthymios Triantafyllou, Konstantinos Drougkas, Georgia Gomatou, Elias Kotteas

**Affiliations:** Oncology Unit, Third Department of Medicine, “Sotiria” General Hospital for Diseases of the Chest, National and Kapodistrian University of Athens, 152 Messogion Avenue, 11527 Athens, Greeceilkotteas@med.uoa.gr (E.K.)

**Keywords:** estrogen receptor, breast cancer, estrogen receptor-low-positive breast cancer, endocrine therapy, triple-negative breast cancer

## Abstract

The expression of estrogen receptors (ERs) in breast cancer (BC) represents a strong prognostic and predictive biomarker and directs therapeutic decisions in early and advanced stages. ER-low-positive BC, defined by the immunohistochemical (IHC) expression of ERs from 1% to 9%, constitutes a distinct subset of total BC cases. Guidelines recommend that a low expression of ERs be reported in pathology reports since the benefit of endocrine therapy in patients with ER-low-positive BC is uncertain. Recently, several cohorts, mostly of a retrospective nature, have been published, reporting the clinicopathological characteristics and outcomes of ER-low-positive BC. However, the majority of the data focus on early-stage BC and the use of (neo)adjuvant therapy, and there is a significant lack of data regarding metastatic ER-low-positive BC. Further factors, including tumor heterogeneity as well as the potential loss of ER expression due to endocrine resistance, should be considered. Including patients with ER-low-positive BC in clinical trials for triple-negative breast cancer (TNBC) might improve the understanding of this entity and allow novel therapeutic approaches. The design and conduction of randomized clinical trials regarding this subgroup of patients are greatly anticipated.

## 1. Introduction

Hormone receptors (HR), including estrogen (ER) and progesterone receptors (PR), are expressed in 70–75% of breast cancer (BC) cases and represent one of the cornerstones that direct the therapeutic decisions for patients with BC both in early and metastatic stages [1,2]. The 2020 update of the recommendations of the American Society of Clinical Oncology/College of American Pathology (ASCO/CAP) defines ER-positive BC as samples with 1% or more of tumor nuclei positive for ER expression by validated immunohistochemistry (IHC). Nevertheless, the recommendations outline that a small subset of patients with ER expression between 1 and 10% should be termed ER-low-positive BC and are not likely to benefit from endocrine therapy (ET) [3]. A lack of consensus exists regarding whether the exact 10% expression should be considered ER-low-positive or ER-positive. Hence, the definition of ER-low expression differs in the literature as either 1 to 9% or 1 to 10% [4].

ER-low BC is associated with interesting biological aspects, but it also poses several challenges regarding its management. A low expression of ERs might be observed de novo, or it might develop in the course of the disease [5,6]. In addition, it should be noted that tumors are heterogeneous entities; therefore, the expression of ERs derived from a specific biopsy might not represent the expression in the whole tumor [5]. The optimal management of patients with ER-low BC in early and metastatic settings has not been defined yet. Several recent studies, mostly of a retrospective nature, have described the features of ER-low BC and assessed the benefit of ET, mainly in an adjuvant setting. In the present review, we attempt to summarize the literature and shed light on the biological and clinical perspectives of ER-low BC.

## 2. Estrogens and ER-Mediated Signaling Pathways

Estrogens, known as female sex hormones, play a crucial role in the development and function of the female reproductive system and secondary sex characteristics. They also affect other systems, such as the cardiovascular, musculoskeletal, central nervous and immune systems [7,8]. Four steroid hormones belong to the estrogen family: estrone, estradiol, estriol and estretrol [7]. Estriol and estretrol are present mainly in the course of pregnancy. Estrone is present during menopause, while estradiol is the predominant form during the reproductive years [9]. Estradiol promotes cell proliferation in the endometrium and mammary gland starting from puberty, while during pregnancy, the dominant forms prepare the mammary gland for milk production [7].

At the cellular level, estrogens act through their receptors, the estrogen receptors a (ERa) and b (ERb), which are part of the nuclear receptor family and are encoded by two different genes, *ESR1* and *ESR2* [7]. Similarly to other nuclear receptors [7], their structure enables them to bind with their ligands but also to DNA and act as transcription factors with the aid of other co-activators and co-repressors [9,10]. The isoforms ERa and ERb are highly similar except for their NH2-terminal domain (NTD), which is involved in gene transcription activation [9].

Estrogens pass through the cellular plasma membrane and interact with their receptors via their ligand binding domain (LBD). From this point, they activate several signaling pathways, which can be divided into genomic and non-genomic based on the ability of the hormone-receptor complex to bind directly to the DNA chain at specific domains, known as estrogen response elements (EREs) [9,11]. Moreover, rapid responses to estrogens have been observed, which do not involve genomic signaling and are known as indirect non-genomic signaling. They are mediated through second messenger production and protein kinase activation pathways, leading to signaling cascades that ultimately regulate gene expression. The most important intracellular cascades involve the phospholipase C/protein kinase C cascade, the mitogen-activated protein kinase (MAPK) pathway, the phosphoinositide 3-kinase (PI3K) pathway and the cyclic adenosine monophosphate (cAMP)/protein kinase A cascade [9,12]. For example, the PI3K/protein kinase B (AKT)/mammalian target of the rapamycin (mTOR) pathway, activated by non-genomic estrogen signaling, has been found to be overactive in up to 70% of BCs and is related to ET resistance after long-term estrogen deprivation [13]. Interestingly, crosstalk between non-genomic and genomic signaling pathways has been described, leading to the regulation of transcription factors by protein-kinase-mediated phosphorylation [11].

Progesterone is another steroid hormone involved in the proliferation and morphogenesis of the luminal epithelium, primarily through paracrine signaling pathways. Progesterone binds to a nuclear receptor, the progesterone receptor (PR) [14]. It should be noted that PR expression depends on estrogen levels since PR is a target gene of an ER [15]. Although the expression of the PR is routinely assessed in BC, the clinical value of PR expression is not so strongly established as it is for ER expression; rather, the presence of an intact and functionally active ER pathway is implied when the PR is expressed [16].

## 3. ER-Low-Positive BC

### 3.1. Epidemiology and Clinicopathological Characteristics

Although ER-low-positive BC represents a small subset of all patients with BC, it is important to understand its nature to provide tailored and effective treatment [5]. Lately, several studies have been published reporting the prevalence and characteristics of ER-low BC as well as their response to treatment.

The prevalence of ER-low BC varies from 1.6 to 5.1%, as reported in recent large-scale cohorts (Table 1) [4,17,18,19,20,21,22,23,24]. Interestingly, Makhlouf et al. performed a re-evaluation of ER status in cases considered ER-low-positive at initial evaluation and demonstrated that 45% of these tumors were ER-negative with repeated IHC staining, confirmed by in situ hybridization (ISH) and a quantitative polymerase chain reaction (qPCR). In particular, ER-low-positive samples derived from needle core biopsies were enriched with false-positive ER staining [4]. In the same study, they focused on tumors with precisely 10% ER expression. The results revealed that those cases were significantly lower grade and more PR-positive than tumors with an ER expression of 1–9% and did not show a significant difference from tumors with an ER expression of 11–30% [4].

Regarding the characteristics of patients with ER-low BC, a study showed that ER-high-positive, ER-low-positive and ER-negative BC had no statistical difference related to the age of menarche and body mass index kg/m^2^ [25]. Among patients with ER-high-positive BC, there were significantly more white patients compared to ER-low-positive BC (93.9% vs. 82.9%, *p* < 0.05) [25]. Indeed, it appears that a patient’s profile is similar between ER-low and triple-negative breast cancer (TNBC), as reported in a multicenter prospective registry between 2011 and 2019. The study showed that demographic and clinical characteristics, including racial and ethnic distribution—it is well-known that TNBC is more prevalent among the African American race—and the prevalence of germline *BRCA1/2* mutations were not different between the TNBC and ER-low groups [26].

Several studies have investigated the morphological and immunohistochemical characteristics of ER-low-positive BC in relation to ER-negative and ER-high-positive BC. ER-low BCs are more likely to have a ductal phenotype of a higher histological grade compared to ER-high BCs (83.5% vs. 71.4%, *p* = 0.005) [23]. In another study, ER-low-positive cases were associated with larger tumors, higher grades, more necrosis, more stromal tumor infiltrating lymphocytes (sTILs) and a higher pathologic N stage [24]. In particular, regarding sTILs, the ER-low-positive cases were associated with more sTILs than the ER-high-positive cases, whereas no difference was found between ER-low-positive and ER-negative tumors. A further survival analysis demonstrated that higher sTIL levels are associated with reduced mortality in ER-negative and ER-low-positive BC [24]. Cases of BC with 1–9% ER expression are more likely to have a higher Ki-67 index and are more likely to be PR-negative [27,28,29]. Moreover, a recent study demonstrated that HER2-low expression was positively associated with the level of ER expression, and ER-low-positive tumors were enriched among HER2 0–2+ tumors [30].

Additionally, studies with further IHC and molecular analyses have demonstrated that vimentin, the epidermal growth factor receptor (EGFR), CPK5/6, and CK14 are highly expressed in ER-low-positive or negative BC and less expressed in ER-high-positive BC [5]. ER-high-positive BCs are more frequently negative for C-kit, p63 and the androgen receptor (AR) compared to ER-low-positive or ER-negative BC [24]. A decrease in vimentin expression was correlated with an increase in ER expression in an older study [31]. In addition, the expression of the *ESR1* gene has been investigated among cases with ER-low-positive BC. Iwamoto et al. reported that the average *ESR1* expression was significantly increased in the ≥10% ER-positive group compared to the 1% to 9% ER expression or ER-negative groups [32]. Consistent findings were reported in a recent study where the average *ESR1* expression was significantly higher in the ER-high-positive cohort than in the ER-low or negative cohort [19]. However, in another study that evaluated the expression levels of a selected set of ER-regulated genes, namely *ESR1*, *PgR*, *GATA3*, *TFF1*, *FOXA1* and *XBP1* along with a panel of three reference genes, the results demonstrated that the tumors in the ER-low group were almost evenly distributed between the ER-high-positive and negative groups [33]. ER-low BCs are more likely to carry a *BRCA1* or *BRCA2* mutation, and this finding indicates the need for genetic counseling and *BRCA* testing in this subset of patients [34]. High frequencies of *TP53* but not *PIK3CA* mutations have been shown in ER-low-positive BC Furthermore, a recent study investigated the prognostic role of H3 lysine nine trimethylation (H3K9me3) in relation to ER status. ER-positive tumors were stratified by ER-low and ER-high-positive tumors, and the prognostic role of H3K9me3 was significant only among the ER-high-positive patients, indicating distinct pathogenicity among the two groups [35].

### 3.2. Prognosis and (Neo)adjuvant Therapy

Most data on the prognosis of ER-low-positive BC are obtained from retrospective studies mainly involving patients with early BC (Table 2). A large-scale retrospective study from Europe showed that the time to local recurrence, time to lymph node recurrence and time to metastasis among HER2-negative BC were similar in ER-low and ER-negative BC and higher compared to ER-high-positive BC [22]. Notably, in the category of HER2-positive BC, ER-low-positive, ER-negative and ER-high-positive BC did not have significant differences in terms of prognosis. The authors conclude that HER2-negative and concomitantly ER-low-positive BC resemble TNBC [22]. A large cohort from Korea reported consistent findings. In this epidemiological retrospective study, the highest 5-year disease-free survival (DFS) rate was observed in patients in the ER-high/HER2-negative cohort (94.0%), and the lowest 5-year DFS rates were in patients in the TNBC cohort (81.3%) and the ER-low/HER2-negative cohort (85.7%) [21]. The shorter DFS for the TNBC and ER-low/HER2-negative combined cohorts were significantly correlated with higher tumor stage, lymphovascular invasion, greater regional lymph node involvement, and larger tumor size [21]. The patients with ER-low BC had a statistically significant worse DFS and overall survival (OS) compared with patients with ER-positive BC, whereas no differences were reported between the ER-low and ER-negative subgroups in a meta-analysis of retrospective studies that included patients with BC who received neoadjuvant chemotherapy (NAC) [36]. However, it should be noted that a recent study from Norway that included women diagnosed with BC in 1995 or later demonstrated that the cumulative risk of death from BC was 22.3% after five years for ER expression < 1% and 8.3% for both the ER-low-positive and ER expression ≥ 10% groups, meaning that there was no apparent difference in the risk of death from BC between the ER-low-positive and ER expression > 10% groups [37].

An important and relevant question is whether adjuvant ET confers survival benefits in patients with ER-low-positive BC. In 2011, the Early Breast Cancer Trialists’ Collaborative Group conducted a patient-level meta-analysis aiming to associate the levels of ER expression with the recurrence reduction with the use of 5-year adjuvant tamoxifen [38]. The results showed a significant benefit in the subgroup analysis even for patients with marginally ER-positive BC (10–19 fmol/mg cytosol protein) from tamoxifen (risk ratio ± standard error, 0.67 ± 0.08) [38]. Nevertheless, several recent retrospective studies have not confirmed this finding. In a retrospective study of 9639 patients with early BC, it was reported that (a) no significant difference was observed in recurrences between patients with ER-low and ER-negative tumors (19.4%) (*p* = 0.5), (b) for patients receiving ET, recurrence rates were higher in patients whose tumors were ER-low-positive compared with those that were ER-positive with ER expression ≥ 10% (17.7% versus 7.7%, *p* = 0.02) and (c) there was no significant difference in total recurrences between the groups of patients who did not receive ET [39]. Another study showed that the 5-year DFS and OS did not significantly differ between ER-negative and ER-low-positive groups, irrespective of receiving endocrine treatment [40]. A lack of benefit from ET in patients with ER-low BC has recently been shown in a meta-analysis, including more than 16,000 patients. This meta-analysis indicated that patients with early BC and ER expression between 1 and 9% gained no significant survival benefit from ET but exhibited a better overall prognosis than patients with ER expression < 1% [41]. Nevertheless, a recent study demonstrated that ET was correlated with increased breast cancer-specific survival in patients with ER-low BC. No significant difference in breast cancer-specific survival was observed between patients who received 2–3 years and >3 years of ET [42]. The potential of a de-escalation strategy was also suggested in a recent propensity-matched analysis, which reported that there was no significant difference in DFS between patients who received 2–3 years and five years of ET (HR, 0.82; 95% CI, 0.51–1.33; *p* = 0.43), indicating that short-term ET for 2 to 3 years might be an alternative for patients who have ER-low-positive BC [43].

In early-stage ER-positive BC, the decision to offer adjuvant chemotherapy depends on the risk of recurrence, which is assessed with clinicopathological criteria and genomic tests [1]. Assuming that a case of ER-low-positive BC is diagnosed in an early stage, without lymph nodes or with minimal node involvement (1–3 lymph nodes), it is reasonable to ask whether using genomic tests is of the same utility as for ER-high BC [44]. A recent study evaluated the role of the Oncotype Dx Breast Recurrence Score Assay in 38 patients with ER-low-positive BC [45]. The results revealed that the majority of the patients with HER2-negative/ER-low-positive BC had a recurrence score (RS) > 25, and the authors concluded that perhaps genomic tests are of limited use as most patients are likely to benefit from adjuvant chemotherapy [45].

Furthermore, NAC therapy is sometimes indicated in early ER-positive BC in order to downstage the tumor; however, it is well known that patients with ER-positive BC are not likely to achieve a pathologic complete response (pCR), contrary to patients with TNBC and HER2-positive disease. It has been reported that the pCR rate of patients with ER-low BC was intermediate between the pCR rate of patients with ER-high and ER-negative BC following NAC treatment [46]. In another study, among 358 patients receiving NAC, the pCR rates were similar for the TNBC and ER-low-positive groups (49.2% vs. 51.3%, respectively, *p* = 0.808) [26]. Moreover, in a cohort of 165 patients that received NAC, the pCR rate was comparable between the two groups (38% in the ER-negative group, 44% in the ER-low-positive group, *p* = 0.498) [47]. Interestingly, Fujii et al. identified 9.5% ER expression as the cut-off percentage below which a pCR was likely [48]. Additionally, when comparing ER-negative, ER-low, and ER-high-positive BC in NAC clinical trial cohorts (*n* = 2765), the results demonstrated no significant differences in the pCR rates between women with ER-low-positive tumors and women with TNBC [49]. In general, the significant pCR rates in TNBC cases are attributed to the higher cell proliferation rates compared to ER-positive BC [50]. The addition of immunotherapy has also increased the rates of pCR in TNBC, which is mainly relevant for the immunogenic subtypes of the disease [50]. It has been suggested that patients with ER-low and HER2-negative BC could be included in the clinical trials of NAC for TNBC and potentially share the same benefit from the addition of immunotherapy, as discussed below [50].

### 3.3. Immune Microenvironment and Immunotherapy

Given the remarkable advances in the field of oncology immunotherapeutics, particular interest lies in the potential of immunotherapy in BC. In general, ER-negative tumors are characterized by increased sTIL infiltration, CD8 + T-cells, and a higher expression of immune-related gene sets, resulting in a more inflamed tumor microenvironment, while ER-positive BC is traditionally considered to be an immunologically “cold” tumor [51,52].

The immunological features of HER2-negative BC with low-positive (1–9%) or intermediate-positive (10–50%) ER expression were investigated in a recent study, as compared to TNBC and tumors with high ER expression (>50%) [53]. The results showed that among the groups of BC with an ER expression of 0%, an ER expression of 1–9% and an ER expression of 10–50%, the levels of stromal TILs, CD8 + T cells and PD-L1 positivity were similar [53]. Also, the expression of certain immune-related gene signatures in tumors with an ER expression of 1–9% and an ER expression of 10–50% was analogous to an ER expression of 0% and higher than in tumors with an ER expression of 51–99% and an ER expression of 100% [53]. Although there is currently no data on patients with ER-low BC who received immunotherapy in early or metastatic settings, since ER-low BC biologically mimics TNBC, it has been suggested that those patients could be included in clinical trials of TNBC and potentially derive benefit from immunotherapy [50]. However, it should be noted that TNBC exhibits a great degree of heterogeneity and includes several phenotypes, not all of which are immunogenic [50]. The presumed biological similarities between ER-low-positive BC and TNBC might be limited to particular phenotypes of TNBC and need to be further explored.

## 4. Knowledge and Research Gaps in ER-Low-Positive BC (Figure 1)

### 4.1. Early-Stage ER-Low-Positive BC

Accumulating evidence has been published questioning the benefit of adjuvant ET for patients with ER-low-positive BC; however, the data remain contradictory [38,39,41,42]. The retrospective nature of the majority of the studies, the heterogeneous design and the different endpoints limit the drawing of clear conclusions. Notably, at the 17th St. Gallen International Breast Cancer Consensus in 2021, the panel was dichotomized on the optimal ER threshold for endocrine therapy initiation [54]. The duration of ET could also be discussed, with some studies suggesting an alternative option with short-term adjuvant ET [42,43].

Besides adjuvant ET, numerous questions arise concerning the following: (a) when should NAC therapy be proposed for patients with ER-low BC and which is the optimal regimen; (b) should the majority of the patients with ER-low BC receive adjuvant chemotherapy; and (c) what is the role of adjuvant cyclin-dependent kinase (CDK)4/6 inhibitor therapy, which has been recently introduced in high-risk patients with ER-positive BC [44]. The stratification of patients according to ER status, including the ER-low-positive group, in randomized clinical trials might improve the understanding of those questions. In parallel, the introduction of patients with ER-low BC in clinical trials of TNBC may illustrate better tactics for their management. A recent phase II trial (NeoPACT) assessing the addition of pembrolizumab in carboplatin plus docetaxel in patients with TNBC allowed for the inclusion of patients with ER-low BC, who comprised 15% of the study population [55].

### 4.2. Metastatic ER-Low-Positive BC

There is a significant lack of published real-world cohorts regarding patients with metastatic ER-low-positive BC. The combination of a CDK 4/6 inhibitor plus ET, the current standard of care for ER-positive BC, is theoretically indicated in these cases [2]. However, should these patients be assumed to be mostly endocrine-resistant and more chemo-sensitive? In parallel, the introduction of immunotherapy for metastatic TNBC raises the question of the potential benefit to the biologically similar ER-low-positive BC.

The latest European School of Oncology/European Society of Medical Oncology (ESO/ESMO) consensus guidelines recommend that the 2020 ASCO/CAP acknowledgment that patients with tumors with ER staining between 1% and 10% represent a new reporting category with proximity to ER-negative BC, without solid data concerning the benefit from ET, should also be adopted for patients with metastatic BC with a low ER-positive status [56]. In particular, the guidelines state that patients with ER-low-positive and HER2-negative metastatic BC should not be considered for ET exclusively and could be considered patients with TNBC for clinical trials [56].

### 4.3. ER Expression Heterogeneity

The identification of low ER expression in a single biopsy might not reflect the expression pattern of the whole tumor mass(es). It has been observed that BC exhibits a degree of genotypic and phenotypic heterogeneity, which could be distinguished into intertumor and intratumor heterogeneity [57]. The expression of ERs could be different between primary and metastatic lesions or in different parts of the same tumor. This phenomenon cannot be currently encompassed in a single pathology report, especially when the biopsy is small [58].

For example, the hormone receptors’ conversion in metastatic BCs, either from a positive primary tumor to a negative metastasis or the opposite, has been reported as high as 18.3% for ERs and 40.3% for the PR [59]. Such discordance could mislead the selection of an effective therapy, especially when only one biopsy is available and it may not reflect the phenotype of the whole tumor [58,60]. The latest guidelines recommend considering the use of ET whenever ER expression is positive in at least one biopsy, even in cases of discordance between ER expression in primary and metastatic samples [56]. Identifying and quantifying the heterogeneity is of utmost importance, as it has a significant role in deciding the suitable therapy and predicting the outcome [57]. Perhaps the development, validation and incorporation of liquid biopsies could bypass this obstacle and lead to optimal therapeutic decisions [61,62].

### 4.4. ER Loss Due to Endocrine Resistance

Endocrine resistance, either primary or secondary, is a major challenge that could occur during the therapy of ER-positive BC [63]. It has been proposed that a proportion of ER-negative and ER-low-positive cells stem from ER-positive cells that lose their ER expression [64]. This alteration could happen spontaneously, due to the selective pressure caused by the absence of estrogen, or even as an adaptive response against specific pharmacological agents [64]. More specifically, it has been shown that a loss of ER expression occurs in approximately 10–20% of the cases during disease progression [63].

The mechanisms involved in the suppression of ER expression include genetic or epigenetic changes in the *ESR1* gene, post-translational modifications or altered receptor tyrosine kinase signaling and cell cycle regulation [63,65,66]. Perhaps the identification of ER-low-positive BC during the disease course could be attributed to ER loss due to endocrine resistance. With an “out-of-the-box” approach, mainly in the pre-clinical research field, we could assume that the finding of ER-low positivity might not preclude ET but rather guide a strategy aiming to reverse this process and re-sensitize the tumor to ET [64].

**Figure 1:**
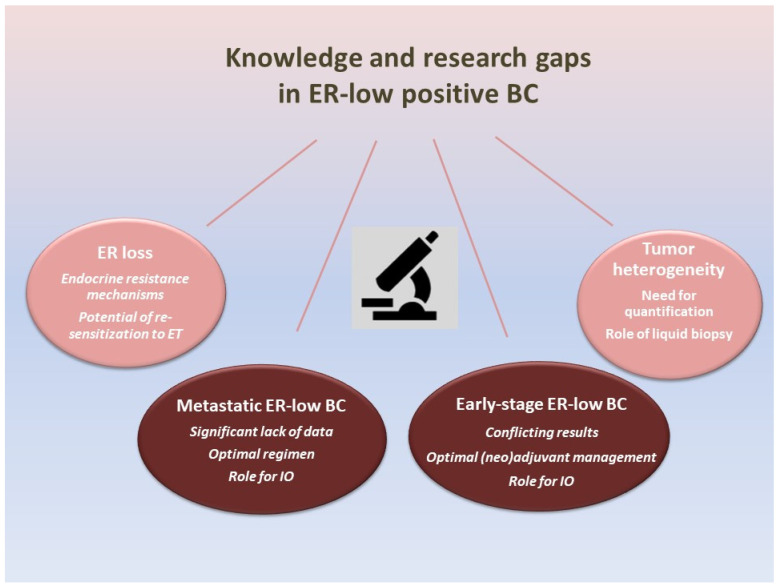
The figure illustrates the research and knowledge gaps regarding ER-low-positive BC. Knowledge and research gaps regarding ER-low-positive BC include the interpretation of conflicting data on early-stage ER-low-positive BC, the significant lack of data on metastatic ER-low BC and incorporating aspects of ER heterogeneity and ER loss due to endocrine resistance. ER: estrogen receptor; BC: breast cancer; ET: endocrine therapy; IO: immunotherapy.

## 5. Conclusions

ER-low-positive BC comprises a small subgroup of the total BC cases but represents a challenging entity with unclear management. Given the conflicting results leading to uncertainty in clinical practice, the role of biomarkers for predicting the benefit of different therapies should be evaluated, including the expression of PR, HER2 or other immune-related biomarkers, such as the sTILs. The inclusion of those patients in clinical trials for TNBC might provide valuable information regarding better management options; however, the significant heterogeneity of TNBC should be taken into account. Finally, well-designed randomized clinical trials for this well-characterized population are greatly anticipated.

## Figures and Tables

**Table 1:** The prevalence of ER-low-positive breast cancer.

Author (Year)	*N*	Prevalence of ER-Low BC	Reference
Makhlouf (2023)	7559	1.6% (123/7559)	[4]
Moldoveanu (2023)	232,762 ^a^	2.0% (4584/232,762)	[17]
Li (2023)	9082	3.29% (299/9082)	[18]
Luo (2022)	5466 ^b^	5.1% (277/5466)	[19]
Yoon (2022)	2162 ^b^	2.5% (54/2162)	[20]
Park (2021)	5930 ^b^	2.0% (117/5930)	[21]
Schrodi (2021)	38,560 ^b^	2.0% (861/38,560)	[22]
Fei (2021)	4179	2.3% (97/4179)	[23]
Poon (2020)	1824	3% (54/1824)	[24]

^a^ only HER2 (–) ^b^ only early breast cancer cases. ER: estrogen receptor, BC: breast cancer, HER2: human epidermal growth factor receptor 2.

**Table 2:** Recent studies on prognosis of patients with early-stage ER-low BC.

Author (Year)	Type of Study	Results	Reference
Schrodi (2021)	Retrospective population-based cohort study	Significantly decreased OS of ER-low/HER2(–) compared to ER-positive/HER2(–)	[22]
Park (2021)	Retrospective unicentric cohort	DFS and OS in the ER-low/HER2(–) cohort were more similar to the TNBC cohort than those with ER-high/HER2(–) BC	[21]
Paakkola (2021)	Meta-analysis	Significantly worse DFS and OS of ER-low patients compared to patients with ER-positive BC	[36]
Skjervold (2023)	Retrospective population-based cohort study	No significant difference in prognosis (risk of death from BC) of patients with ER-low BC compared to those with ER-positive BC for patients diagnosed after 1995	[37]

OS: overall survival; ER: estrogen receptor; HER2: human epidermal growth factor 2; DFS: disease-free survival; TNBC: triple-negative breast cancer; BC: breast cancer.

## Abbreviations

AKT: Protein Kinase B; AR: Androgen Receptor; ASCO: American Society of Clinical Oncology; BC: Breast Cancer; BRCA1/2: Breast Cancer gene 1/2; cAMP: cyclic Adenosine Monophosphate; CAP: College of American Pathology; CDK: Cyclin-dependent Kinase; CK14: Cytokeratine 14; CPK 5/6: Creatine Phosphokinase; DFS: Disease-Free Survival; DNA: Deoxyribonucleic Acid; EGFR: Epidermal Growth Factor Receptor; ER: Estrogen Receptor; ERE: Estrogen Response Elements; ESMO: European Society of Medical Oncology; ESO: European School of Oncology; ESR1: Estrogen Receptor 1; ET: Endocrine Therapy; FOXA1: Forkhead Box A1; HER2: Human Epidermal Growth Factor Receptor 2; HR: Hormone Receptor; IHC: Immunohistochemistry; IO: Immunotherapy; ISH: In Situ Hybridization; LBD: Ligand-Binding Domain; MAPK: Mitogen-Activated Protein Kinase; mTOR: mammalian Target Of Rapamycin; NAC: Neoadjuvant Chemotherapy; NTD: NH2-Terminal Domain; OS: Overall Survival; P63: Protein 63; pCR: pathologic Complete Response; PD-L1: Programmed Death-Ligand1; PgR: Progesterone Receptor; PI3K: Phosphoinositide-3 Kinase; PIK3CA: Phosphatidylinositol-4,5-Bisphosphate 3-Kinase Catalytic Subunit Alpha; H3K9me3:H3 Lysine 9 Trimethylation; PR: Progesterone Receptor; qPCR: quantative Polymerase Chain Reaction; RS: Recurrence Score; sTILs: stromal Tumor Infiltrating Lymphocytes; TFF1: Trefoil Factor 1; TILs: Tumor-Infiltrating Lymphocytes; TNBC: Triple-Negative Breast Cancer; TP53: Tumor Protein 53; XBP1: X-box Binding Protein 1.
